# Reverse-Bumpy-Ball-Type-Nanoreactor-Loaded Nylon Membranes as Peroxidase-Mimic Membrane Reactors for a Colorimetric Assay for H_2_O_2_

**DOI:** 10.3390/s16040465

**Published:** 2016-04-01

**Authors:** Ying Tong, Xiangyu Jiao, Hankun Yang, Yongqiang Wen, Lei Su, Xueji Zhang

**Affiliations:** Research Center for Bioengineering and Sensing Technology, School of Chemistry and Biological Engineering, University of Science and Technology Beijing, Beijing 100083, China; gailty2004@163.com (Y.T.); jiaoxiangyu@126.com (X.J.); ppyhk@hotmail.com (H.Y.); wyq_wen@ustb.edu.cn (Y.W.)

**Keywords:** nanoreactor, flexible membrane reactor, peroxidase-mimic membrane reactor, gold nanoparticles, polydopamine

## Abstract

Herein we report for the first time fabrication of reverse bumpy ball (RBB)-type-nanoreactor-based flexible peroxidase-mimic membrane reactors (MRs). The RBB-type nanoreactors with gold nanoparticles embedded in the inner walls of carbon shells were loaded on nylon membranes through a facile filtration approach. The as-prepared flexible catalytic membrane was studied as a peroxidase-mimic MR. It was found that the obtained peroxidase-mimic MR could exhibit several advantages over natural enzymes, such as facile and good recyclability, long-term stability and easy storage. Moreover, the RBB NS-modified nylon MRs as a peroxidase mimic provide a useful colorimetric assay for H_2_O_2_.

## 1. Introduction

Reverse bumpy ball (RBB)-type nanoreactors are rapidly attracting increasing interest [1,2]. The term RBB refers to a hollow porous sphere in which numerous nanoscale-sized catalysts remain supported on or partially embedded in the inner walls of the shell, in contrast to the yolk-shell structure [1]. RBB-type nanoreactors have been considered to offer additional benefits with respect to the yolk-shell countparts. For instance, the RBB-type nanoreactors can provide a greater quantity of catalytically active sites per nanoreactor [3,4]. Moreover, due to the higher contact between the catalysts and the support shell, potential synergistic effects between the catalysts and the supports may be more efficiently exploited [1,5]. Due to the distinctive features of the RBBs, synthesis of various RBBs and relative applications have been pursued in recent years. Nanoparticle catalysts (*i.e.*, Au [6,7], Pd [4,8,9], Pt [3,10,11], Mn_3_O_4_ [12], and Fe_3_O_4_ [13]) have been attached to the inner walls of various types of porous spheres including silica [3,4,12] CeO_2_ [9,11] carbon [8] and polymers [7], achieving high catalytic activity.

However, the recyling process of RBB-type nanoreactors in liquid media is tedious and often laborious as a result of the required isolation by centrifugation/sedimentation or filtration [4,14,15], which has hampered the recovery and reusability of the RBB-type nanoreactors in liquid media. Compared with the dispersion of catalysts in solution, thin film-type catalysts possess more favorable properties from a practical viewpoint. For instance, switching the reaction off or on through thin film catalysts is technically easier to realize, just behaving like a “dip catalyst” [16,17,18,19]. In addition, it is easier to separate thin film catalysts from reaction solutions, offering the feasibility and ease of multiple reuse [16,17,18,19]. On the other hand, recently, fabrication of flexible membrane reactors (MRs) has been pursued partly because the gained flexibility can allow the construction of catalytic reactors with arbitrary geometries [20,21,22], and great attention has been paid to preparing catalytic films on porous flexible substrates. Among common flexible materials, nylon membranes are particularly attractive [20,21] due to their unique merits such as toughness, high tensile strength, elasticity, and high resistance to acids and alkalis, as exemplified by a recent study by List and co-workers, in which nylon fabric was used as a support for preparing versatile organotextile catalysts [22].

In the present study, we report fabrication of RBB-type-nanoreactor-based flexible membranes. Template carbonization method is used to synthesize the RBB-type nanoreactors. Then, the RBB-type nanoreactors are loaded on flexible nylon membranes through a facile filtration approach. The as-prepared flexible catalytic membrane as the peroxidase-mimic membrane reactors is studied. Furthermore, it is tested as a H_2_O_2_ sensor.

## 2. Results and Discussion

**Figure 1:**
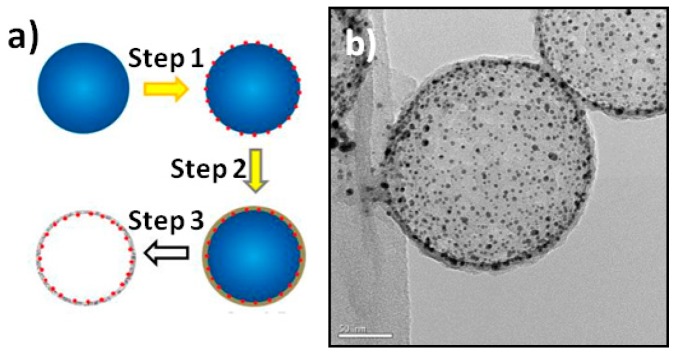
(**a**) Synthetic route to the Au NPs-embedded RBB-structured NSs. Step 1: Au loading; Step 2: PDA coating; Step 3: calcination and the core removal by alkaline etching; (**b**) TEM image of the RBB-structured NSs. Scale bar: 50 nm.

Figure 1a illustrates the synthesis process of the RBB-type nanoreactors. In Step 1, SiO_2_ nanospheres (NSs) were first modified with 3-aminopropyltrimethoxysilane to introduce amine groups on their surface, serving as the sacrificial core. Then, negatively charged Au NPs were deposited on the amino-functionalized SiO_2_ NSs through the electrostatic interactions. In Step 2, the C precursor layers were coated on the surface of the SiO_2_@Au NSs by the self-polymerization of dopamine, forming the SiO_2_@Au@polydopamine sandwich configuration. In Step 3, the as-obtained product was calcined in N_2_ atmosphere to carbonize the PDA shell [17,23] and, finally, the SiO_2_ cores were removed by 2 mol/L NaOH etching for 48 h. The morphology of the final products was characterized by transmission electron microscope (TEM). As shown in Figure 1b, the final products exhibited the characteristic morphology of the RBB configuration: hollow NSs with NPs embedded in the inner walls of the shell.

**Figure 2:**
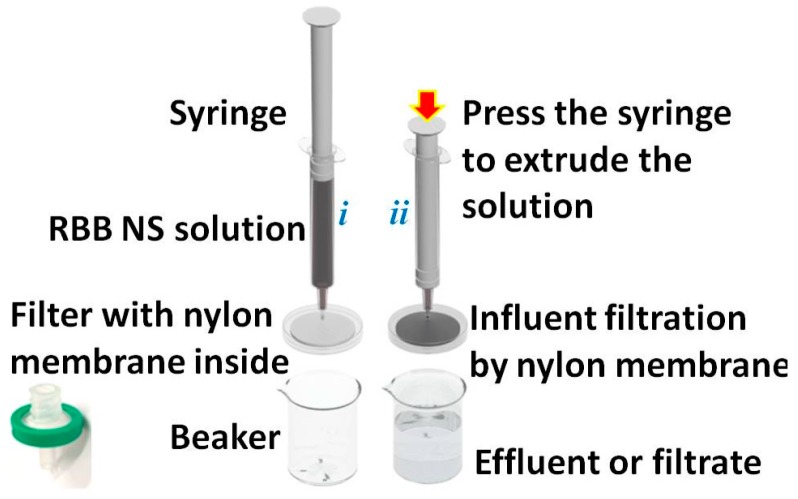
Schematic illustration of the filtration device (*i*) and the filtration process (*ii*).

Figure 2 shows a schematic illustration of the filtration-based fabrication process of the RBB-structured NSs-based catalytic flexible membrane. As illustrated, a piece of 0.20 μm pore nylon membrane was inside in the filter and when the RBB NS-containing solution in the syringe was filtered through the nylon membrane, the RBB NSs were trapped within the nylon membrane, forming the RBB NS-modified nylon membrane.

**Figure 3:**
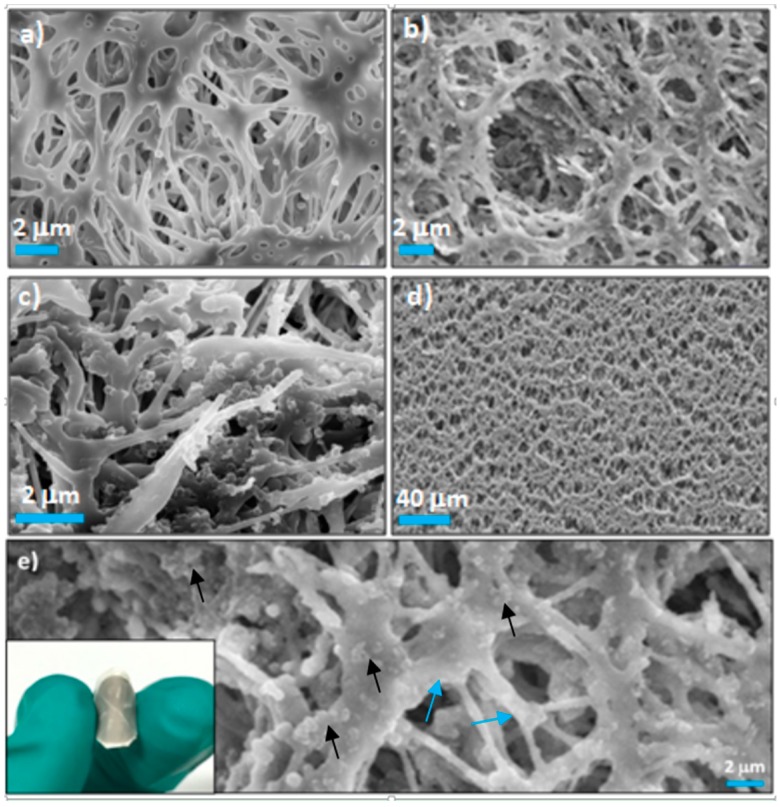
SEM images of the nylon (**a**) and the RBB-structured NS-modified nylon membranes (**b**–**d**); (**e**) High magnification SEM image of the RBB-structured NS-modified nylon membrane. The black arrows indicate the RBB-structured NS, and the blue arrows indicate the nylon fibre network. Inset of (**e**): The flexibility of the RBB-structured NS-modified nylon membrane.

Success in fabrication of the RBB NS-modified nylon membrane is indicated by the visual color change of the membrane from the white before filtration to the black after filtration and by the colorless filtrate. From the plane-view scanning electron microscope (SEM) images of bare nylon membrane and the RBB NS-modified nylon membrane, shown in Figure 3a,b, respectively, it can be seen that the surface of the nylon membrane was modified with the RBB NSs. Cross-section SEM images of the RBB NS-modified nylon membrane (Figure 3c) also indicate that the RBB NSs were introduced into the nylon membrane. A low magnification SEM image of the RBB NS-modified nylon membrane indicating that large-area RBB NS-modified nylon membrane could be obtained is shown in Figure 3d. Furthermore, a high-magnification SEM image of the RBB NS-modified nylon membrane (Figure 3e) reveals that the RBB NSs should be intercepted by small pores of the nylon filter during filtration. Nylon is naturally hydrophilic and has an open pore structure, facilitating the flow of the influent through the membrane. Meanwhile, the nylon membrane is a depth filter and can retain effectively particles larger than 0.20 μm. As a result, the RBB NSs can be effectively immobilized within the nylon membrane. In addition, the resulting RBB NS-modified nylon membrane is not compact and still maintains the open pore structure of the nylon membrane. These structure features of the RBB NS-modified nylon membrane are favorable for mass transfer in catalytic application. Moreover, the photograph (inset of Figure 3e) shows the RBB NS-modified nylon membrane is highly flexible, with no observed change after repeated flexion.

**Figure 4:**
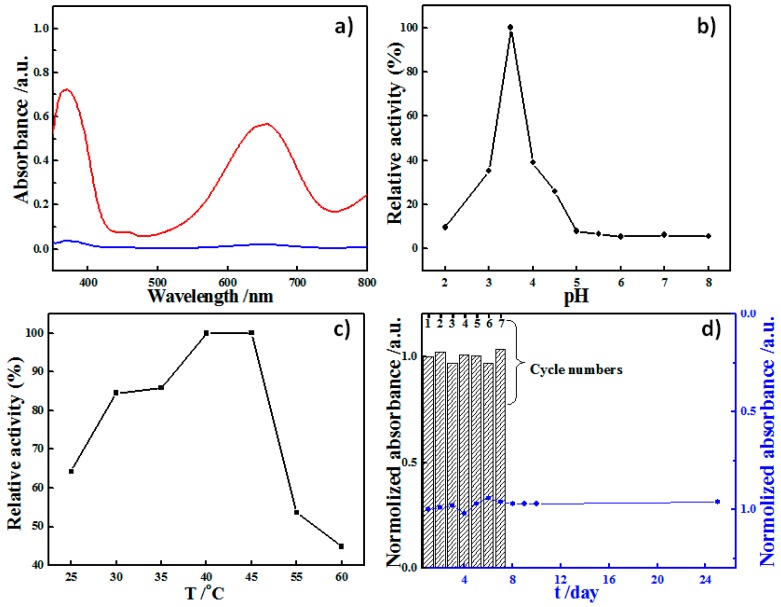
(**a**) UV-vis absorption spectra of the TMB-H_2_O_2_ mixture (0.8 mmol/L TMB, 50 mmol/L H_2_O_2_) in the absence (blue line) and presence of the RBB NS-based MR (red line) after 10 min incubation; (**b**,**c**) Plots of the peroxidase-like activity of the RBB NS-based MR against pH and temperature; (**d**) The recyclability of the RBB NS-based MR indicated by the normalized absorbance of the TMB oxidized the catalytic oxidation of 0.8 mmol/L TMB by 10 mmol/L H_2_O_2_ in seven successive cycles with the same MR (upper) and the long-term stability of the catalytic activity of the RBB NS-based peroxidase-like MR (bottom).

To investigate the peroxidase-like activity of the RBB NS-modified nylon membrane, the catalytic oxidation of 3,3,5,5-tetramethylbenzidine (TMB), a benign and noncarcinogenic color reagent, in the presence of H_2_O_2_ was tested. As shown in Figure 4a, the RBB NS-modified nylon membrane could catalyze the oxidation of TMB in the presence of H_2_O_2_ and produce a deep blue color, with maximum absorbance at 650 nm [24]. A kinetic study showed that the RBB NS-modified nylon membrane exhibited its highest catalytic activity at approximately pH 3.5 (Figure 4b). In addition, also like natural enzymes, the peroxidase-mimic catalytic activity of the RBB NS-modified nylon membrane was dependent on temperature, showing a maximum at approximately 40 °C (Figure 4c). But unlike natural enzymes, the RBB NS-based peroxidase-mimic MR could exhibit facile and good recyclability and long-term stability. The recyclability of the RBB NS-based peroxidase-mimic MR was examined by recycling the same MR. Between each cycle, the membrane was directly withdrawn from the TMB-H_2_O_2_ reaction solution and rinsed with deionized water. As revealed from Figure 4d (bars), the MR retained almost unchanged catalytic activity towards TMB oxidation by H_2_O_2_ in seven successive cycles, indicating good recyclability of the RBB NS-modified nylon MR. The peroxidase-mimic activity stability test was further investigated by testing the peroxidase-mimic membrane every day. When not in use, it was stored without any other specific care at room temperature. From Figure 4d (blue dotted line), it can be seen that the MR could maintain a stable catalytic activity for at least 25 d.

**Figure 5:**
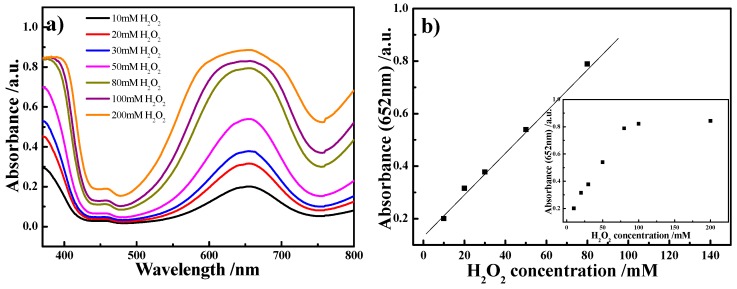
(**a**) Typical absorption spectrum of the TMB solution in the presence of H_2_O_2_ at various concentrations using the RBB NS-modified nylon MRs as a peroxidase mimic; (**b**) Linear calibration plot between the absorbance at 652 nm and concentration of H_2_O_2_. The insert shows the dependence of the absorbance at 652 nm on the concentration of H_2_O_2_ in the range 10 mmol/L to 200 mmol/L.

Furthermore, the catalytic activity of the RBB NS-modified nylon MR is H_2_O_2_ concentration dependent. As shown in Figure 5a, the absorbance of this system increased with increasing H_2_O_2_ concentration. Therefore, the RBB NS-modified nylon MR can be used as H_2_O_2_ sensor, which has potential applications in biomedical fields [25,26]. As shown in Figure 5b, under the optimal conditions (*i.e.*, 40 °C, pH 3.5), the absorbance at 652 nm was proportional to H_2_O_2_ concentration from 10–80 mmol/L with a detection limit of 0.8 mmol/L.

## 3. Experimental Section

### 3.1. Chemicals

Tetraethyl orthosilicate (TEOS), (3-aminopropyl)triethoxysilane (APTS) and hydrogen tetrachloroaurate(III) hydrate were purchased from Alfa Aesar (Ward Hill, MA, USA). Dopamine chloride and 3,5,3’,5’-tetramethylbenzidine (TMB) were purchased from Sigma (St Louis, MO, USA). All other chemicals of at least analytical reagent grade were obtained from Sinopharm Chemical Reagent Co., Ltd. (Beijing, China). Nylon filters (0.20 μm) were purchased from Lanyi Chemical Reagent Co., Ltd. (Beijing, China). Aqueous solutions were prepared using deionized water produced by a Milli-Q water system (Millipore, Darmstadt, Germany).

### 3.2. Synthesis of the RBB-Structured NSs

SiO_2_ NSs (*ca*. 200 nm in diameter) were synthesized according to the Stӧber method. For preparation of amino-functionalized silica NSs, 100 mg silica NSs were dispersed in toluene, and the solution was stirred vigorously after adding 25 μL APTS (0.1 μmol/L). Afterwards, the precipitates were collected by centrifugation, washed three times with ethanol, and then dried overnight under vacuum at 60 °C. Gold NPs (*ca*. 4.5 nm in diameter) were synthesized according to previous report [27]. To prepare the SiO_2_@Au composite NSs, 20 mg amino-functionalized silica NSs were added to the above gold NP solution with stirring for 30 min, followed by centrifugation and drying overnight under vacuum at 60 °C. The obtained SiO_2_@Au NSs were added into the freshly prepared 1 mg/mL dopamine tris buffer (10 mmol/L, pH 8.5) for polymerization of dopamine [28,29]. The mixture was stirred for 2 h, followed by centrifugation, washing with deionized water and drying in vacuum at 60 °C overnight. The obtained powders were carbonized under N_2_ atmosphere at 500 °C for 3 h with a heating rate of 5 °C·min^−1^. Afterwards, the powders were treated with 2 mol/L NaOH solution for 48 h to remove the SiO_2_ core, producing the final product, the RBB-structured NSs.

### 3.3. Preparation of the Flexible Catalytic Membranes

RBB NS-containing solution (1 mL, 0.05 mg/mL) was transferred to a syringe and filtered with a nylon filter membrane. Then, the nylon filter membrane was washed with deionized water and ethanol, followed by drying at 60 °C under vacuum.

### 3.4. Instruments and Measurements

The morphologies of the samples were observed using a field emission scanning electron microscopy (SEM, Supra 55, Zeiss, Oberkochen, Germany) and a field emission transmission electron microscopy (TEM, JEM-2100F, JEOL, Tokyo, Japan). For UV-vis absorption measurements, quartz microcuvettes with 10 mm path lengths and 1 mm window widths were used on a UV-vis spectrophotometer (UV-1800, Shimadzu, Tokyo, Japan). For measurement of TMB oxidation by H_2_O_2_ catalyzed by the RBB NS-based peroxidase-mimic MR, the RBB NS-modified nylon membranes were immersed in NaOAc buffer (25 mmol/L, N_2_ saturation, pH 3.5) containing H_2_O_2_ at different concentrations and 800 μmol/L TMB. The reaction was kept at 40 °C for 10 min. UV-vis absorption spectra were recorded to monitor the time-dependent absorbance changes at 652 nm.

## 4. Conclusions

In conclusion, we have demonstrated the fabrication of a RBB NS-based peroxidase-mimic MR. The obtained RBB-structured NSs could be firmly captured by the nylon membrane by filtration, producing flexible membranes. The obtained catalytic membrane could be used as a peroxidase-mimic MR to catalyze the oxidation of TMB by H_2_O_2_ and exhibited several advantages over natural enzymes such as facile and good recyclability, long-term stability and easy storage. Moreover, the RBB NS-modified nylon MRs as a peroxidase mimic provides a colorimetric assay for H_2_O_2_ with a detection limit of 0.8 mmol/L.
